# Different characteristics of the soil in marmot habitats might be one of the factors that influcting *Yersinia pestis* prevalent in which than pikas

**DOI:** 10.3389/fmicb.2024.1489125

**Published:** 2024-10-18

**Authors:** Wenlong Zhao, Shixiong Li, Yuechen Sun, Jingpeng Liu, Yixin Ma, Rui Qi

**Affiliations:** ^1^School of Public Health, Lanzhou University, Lanzhou, Gansu Province, China; ^2^Jiayuguan Centers for Diseases Control and Prevention, Jiayuguan, Gansu Province, China

**Keywords:** natural plague foci, *Yersinia pestis*, soil characteristics, marmot, pika

## Abstract

**Introduction:**

Marmots are recognized as host animals for plague caused by *Yersinia pestis* infection. It is unclear that why plague prevalent in marmot rather than other rodents like pikas in the same habitats. This study aims to analyze the differences of the soil characteristics around marmots and pikas burrows to explore the soils factors impacting on different epidemic intensities of *Yersinia pestis* in these two rodents.

**Methods:**

Soil samples were collected from within and around marmot and pika burrows, as well as from the nearby areas not inhabited by them and Chinese baseline soil properties as control groups, in the Qilian Mountains of Gansu Province, China. The physicochemical properties and the bacterial 16S rRNA were measured to analyze the characteristics of soils from different groups. Subsequently, the data were analyzed using R studio.

**Results:**

The analysis revealed that marmot habitats exhibited distinct soil characteristics, including lower organic matter and alkaline hydrolyzed nitrogen, but higher electrical conductivity and total soluble salts. And soil in marmot areas tended to have higher concentrations of nickel, chromium, and iron, also lower levels of zinc and selenium. Additionally, the alpha diversity of soil microorganisms in marmot habitats was significantly low. Simultaneously, redundancy analysis was conducted, which showed that the low alpha diversity of marmot-soil was influenced by its physicochemical properties. The alpha diversity of the soil was positively correlated with EC, TSS, Na, and Cr, etc., while it was negatively correlated with AHN, OM, Se, Zn, and Fe, etc.

**Conclusion:**

These characteristics in marmot habitats, including low levels of organic matter, alkaline hydrolyzed nitrogen, zinc, selenium, and bacterial alpha diversity, as well as high levels of electrical conductivity, total soluble salts, iron, and nickel, played a crucial role in the spread of plague. It was discovered that the unique characteristics of marmot-soils provided essential elements necessary for the survival of *Yersinia pestis*, including high levels of Fe and Ca, or facilitated the spread of plague. Thus, the transmission of the plague was facilitated.

## Introduction

Plague, a severe infectious disease caused by *Yersinia pestis*. And its transmission is primarily through flea bites, direct contact with infected animals or humans, and airborne modes (Abdel et al., 2023; Pechous et al., 2016). Historically, several plague pandemics have occurred in different regions around the world (Spyrou et al., 2016). Based on this, it is inferred that the disease may have originated in Central Asia, subsequently spreading throughout Africa and Europe (Sabour et al., 2022). *Yersinia pestis* primarily resides in burrowing wild rodents, such as marmots (Dubyanskiy and Yeszhanov, 2016). Fleas acting as vectors, transmit the bacterium to others by feeding on the blood of these infected hosts (Davis and Isberg, 2014). Regions where these wild rodents live are identified as natural plague foci, provided *Yersinia pestis* can sustain itself in the local natural environment (Andrianaivoarimanana et al., 2013). Such areas are widespread, spanning parts of Asia, the Americas, and Africa. Natural plague outbreaks often occur in regions characterized by continental climates and dry grasslands, which undergo significant weather fluctuations and support large rodent populations. Changes in temperature and precipitation levels can lead to plague outbreaks within these animal populations (Du et al., 2017; Lotfy, 2015). Notably, in northwest China, a large area of natural plague foci exists where human activities intersect with these habitats, and this overlap presents opportunities for the spread of plague (Gao et al., 2010).

The prevalence of plague, particularly within natural plague foci, is influenced by numerous factors, notably the host animals and their parasitic vectors (Nguyen et al., 2018). Marmots, serving as primary hosts for *Yersinia pestis*, are predominantly found in China's plateau regions, making their habitats critical areas for plague monitoring (Xu et al., 2023). Interestingly, some animals frequent in these foci without spreading the disease. The question of why plague is prevalent among marmots, but not other rodent species like pikas, such as pikas, leads to two main perspectives: (1) Parasitic vectors and host susceptibility (Dean et al., 2018). A study indicated that host animals of natural plague foci often harbored more parasitic vectors, and *Yersinia pestis* capable of surviving a period of time in parasitic vectors, facilitating transmission to other animals (Sariyeva et al., 2019). Another study showed that most marmots carried *A. phagocytophilum*, making them more susceptible to *Yersinia pestis* (Duan et al., 2022). However, pikas, which share habitats with marmots and also carry many parasitic vectors, show a negligible presence of *Yersinia pestis* (Brinkerhoff et al., 2020). This discrepancy suggests other factors at play in the bacterium's host preference. (2) Another viewpoint is the soil. Globally, studies on the relationship between soil and the plague have been conducted (Markman, 2019). There is a view that the epidemic of plague was related to the natural changes in ecological conditions and the natural preservation of *Yersinia pestis* in the soil. As early as the Third Plague Pandemic, there has been speculation and debate over the possibility of *Yersinia pestis* existing in soil, thus influencing plague spread (Lynteris, 2017). The interaction between soil and rodents is dual: the physicochemical properties of soil may vary based on rodent habitat selection, and rodent activity can alter these soil properties. Specific soil characteristics may affect the survival rate and longevity of *Yersinia pestis* in the environment (Fleming et al., 2014).

In recent years, the shifting global climate and human activities have led to the identification of new plague host animals and vectors, such as *Spermophilus alashanicus* and *Ochotona daurica*, and vectors like *Neopsylla abagaitui* and *Citellophilus tesquorum mongolicus*, across various regions (Dubyanskiy and Yeszhanov, 2016). Human intrusion into natural plague foci can inadvertently facilitate the spread and outbreak of the disease (Ge et al., 2015). Despite numerous studies on marmot habitats, there is a lack of research examining why the plague predominates in marmots over other rodent species within these foci. The role of habitat factors, particularly soil factors selected by marmots and other rodent species, in influencing plague prevalence remains unclear. Therefore, this study involved measuring the physicochemical properties and microorganisms present in the soil to evaluate whether soil characteristics influencing plague prevalent in marmots than pikas.

## Materials and methods

### Samples collection

Field surveys and sample collections were conducted in a natural plague focus in the Qilian Mountains, where the collection sites were located in alpine grassland ecosystems at the altitude of 2,500 to 3,000 meters. Soil samples included soil from inside and surrounding the burrows of pikas and marmots. Additionally, soil samples of control groups were collected from non-rodent habitats within the natural plague focus, which shared similar ecological environments including altitude, climate, and vegetation, but lacked rodent burrows. During the collection process, impurities such as vegetation, weeds, and stones were removed from the soil as much as possible. Using a shovel, approximately 100 grams of soil from approximately 10 centimeters below the surface was collected inside each marmot and pika burrow. Similarly, soil samples were collected within a 1-meter radius around the burrows. Each soil collection point was at least 20 meters apart from the next to ensure spatial independence. The collected soil samples were individually placed in sealed bags, labeled with corresponding numbers, and relevant information such as the sampling location. The collected soil samples were air-dried in a shaded area, and during the drying process, they were intermittently stirred and impurities were removed. After 1 week, the dried soil samples were ground and sequentially sieved through 2 mm, 10 mesh, and 100 mesh screens. From the collected samples, 30 specimens were ultimately selected for soil testing and analysis: 6 samples from inside marmot burrows (MI), 6 samples from the surrounding area of marmot burrows (MO), 6 samples from inside pika burrows (PI), 6 samples from the surrounding area of pika burrows (PO), and 6 control samples from non-rodent inhabited areas (C).

### Physicochemical properties and microbial detection of soil samples

Soil particle size was measured using a laser particle size analyzer. According to different particle sizes, soil particle sizes could be classified into different types: clay (< 2 μm), silt (2~50 μm), fine sand (50~250 μm), and coarse sand (250~2,000 μm). The physicochemical properties of soil were measured using the following methods: soil pH was determined utilizing the electrode method (NYT 1377-2007, China), while soil electrical conductivity (EC) was assessed by the platinum electrode method (HJ802-2016, China). Total soluble salt (TSS) content in the soil was measured utilizing the weight method (NY/T1121.16-2018, China), and organic matter (OM) content in the soil was determined using the potassium dichromate heating method (LYT1237-1999, China). The soil alkaline hydrolysis nitrogen (AHN) content and available phosphorus (AP) content were analyzed using the Kjeldahl method (LYT128-2015, China) and molybdenum antimony anti-colorimetry (LY_T1232-2015, China), respectively. Flame photometry (LY/T1234-2015, China) was utilized for measuring soil available potassium (AK) content, while ICP-AES (GB15618-2018, China) was used for analyzing 9 metal elements in soil: calcium (Ca), iron (Fe), magnesium (Mg), sodium (Na), zinc (Zn), chromium (Cr), copper (Cu), nickel (Ni), and lead (Pb). Soil metalloid elements, including selenium (Se), arsenic (As), and mercury (Hg), were quantified through atomic fluorescence spectrometry (GB/T22105, China).

High-throughput sequencing of 16S RNA was used for soil microorganisms detection. Total DNA was extracted from the soil samples using the E.Z.N.A™ Mag-Bind Soil DNA Kit. Subsequently, the target gene was amplified using universal bacterial primers, with the primer names and sequences being 341F (CCTAYGGGRBGCASCAG) and 806R (GGACTACNNGGGTATCTAAT). The PCR amplification process comprised two distinct cycling stages, executed under the following conditions: Initially, a denaturation step was conducted at 94°C for 3 min. Then followed by 5 cycles of denaturation at 95°C for 30 seconds, annealing at 45°C for 30 seconds, and extension at 72°C for 30 seconds. Subsequently, 20 cycles of denaturation at 95°C for 30 seconds, annealing at a higher specificity temperature of 55°C for 30 seconds, and extension at 72°C for 30 seconds were performed. And a final extension at 72°C for 5 min. The PCR products were then analyzed using 2% agarose gel electrophoresis to assess the library size. Furthermore, the DNA concentration of each PCR product was determined using the Qubit^®^ 4.0 Green dsDNA Assay Kit, and a bioanalyzer (Agilent 2100, USA) was utilized for quality control. Free primers and primer dimers were purified from the amplicon products using Hieff NGS™ DNA selection beads (Yeasen, 10105ES03, China). Sequencing was conducted on the Illumina MiSeq platform (Illumina MiSeq, USA) to obtain the original bacterial sequences. After sequencing, two short Illumina reads were merged, and then the primer sequences from the merged sequences were removed using Vsearch software (version 2.8.1). Effective tags were clustered into Operational Taxonomic Units (OTUs) with a similarity threshold of ≥97%. Chimeric sequences and singleton OTUs (with only one read) were excluded. The remaining sequences were then assigned to each sample based on OTUs. Taxonomic annotation was carried out using a bacterial taxonomy database to identify the microbial community composition.

### Statistical analysis

Alpha diversity was assessed using the Simpson and Shannon indices, while beta diversity was evaluated based on Bray–Curtis distances and visualized through a principal coordinate analysis (PCoA) plot. Redundancy analysis was also used to determine the relationship between soil physicochemical properties and bacterial abundance. Values were expressed as the mean ± standard error of the mean (SEM). Comparison of data between different groups were performed using *t-*test, and *p*-value of < 0.05 was considered statistically significant. Data analysis and plotting were performed using R software (version 4.3.3).

## Results

### Differences in physicochemical properties of soil between different groups

**Figure 1:**
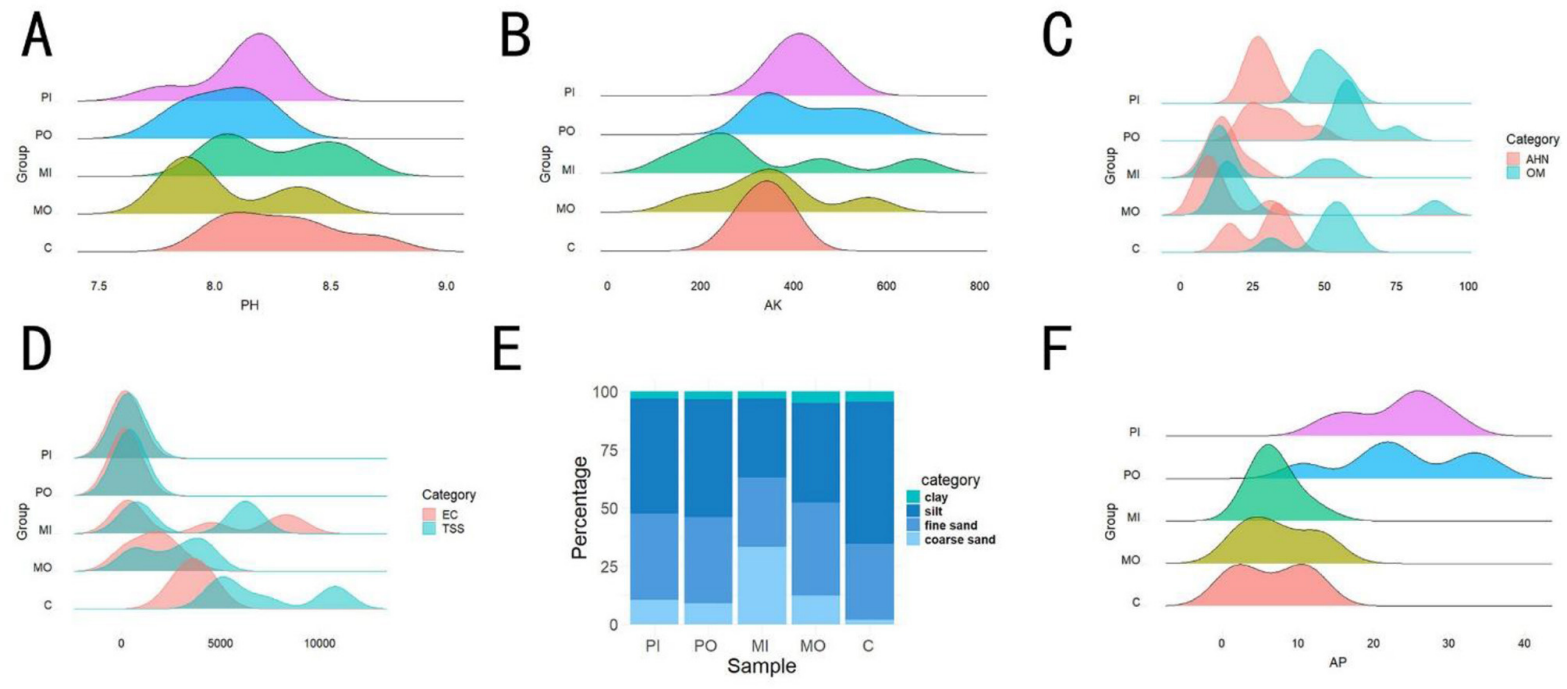
Effects of different rodent species on soil physicochemical properties. **(A)** Differences in soil pH among 5 groups; **(B)** differences in soil available potassium across 5 groups; **(C)** analysis of variations in soil organic matter and alkali hydrolyzable nitrogen among 5 groups; **(D)** analysis of variations in soil electrical conductivity and soil total soluble salts across 5 groups; **(F)** percentage of different soil particle size contents among 5 groups; **(E)** comparison of soil available phosphorus among different groups.

This study presented the average values of various physicochemical properties of soil from 5 distinct groups (Table 1). Due to the extensive range of soil physicochemical properties indicators, the data were divided into two sections for analysis. Initially, wave charts and percentage charts were utilized to analyze the first 8 indicators (Figure 1). It was found that there was no significant difference among the groups for values of the pH and AK indicators (Figures 1A, B). The OM in the soil of marmot habitats exhibited the same trend as AHN, with the levels of soil OM and AHN in the marmot group being approximately twice as low as those in the other groups (Figure 1C). Simultaneously, the TSS and EC values in marmot habitats, particularly within burrows, were slightly lower than those in the control group but significantly higher than in pika-soils (Figure 1D), with the difference being as much as even 10 times. The coarse sand content was highest in marmot burrows, being about 3 times that of the soil outside marmot burrows and pika-soils, while the control soil contained almost no coarse sand (Figure 1E). The contents of AP in marmot habitat soils were similar to those in the control group, both of which were lower than those in pika-soils (Figure 1F).

**Table 1:** Physicochemical properties of samples across different groups.

**Index**	**Group**				
	**PI**	**PO**	**MI**	**MO**	**C**
pH	7.96 ± 0.08	8.04 ± 0.06	8.26 ± 0.09	8.04 ± 0.08	8.29 ± 0.10
TSS (g/kg)	0.73 ± 0.19	0.82 ± 0.15	6.69 ± 2.57	5.50 ± 1.37	14.99 ± 2.11
EC (us/cm)	188.72 ± 16.89	189.53 ± 9.32	3,783.15 ± 1,560.54	1,704.45 ± 528.91	3,619.33 ± 348.20
AP (mg/kg)	23.23 ± 2.35	23.86 ± 3.50	6.29 ± 0.90	7.12 ± 1.76	6.67 ± 1.79
AK (mg/kg)	418.91 ± 23.64	431.90 ± 45.80	331.33 ± 83.92	346.46 ± 58.08	338.04 ± 13.91
Clay (%)	3.31	3.4	3.12	5.1	4.5
Silt (%)	49.22	50.58	33.91	42.72	61.17
Fine sand (%)	37.08	37.07	29.71	39.96	32.51
Coarse sand (%)	10.4	8.95	33.19	12.23	1.83
AHN (g/kg)	0.27 ± 0.01	0.32 ± 0.04	0.16 ± 0.03	0.13 ± 0.04	0.289 ± 0.04
OM (g/kg)	50.40 ± 2.13	61.97 ± 3.16	25.88 ± 7.03	29.89 ± 11.69	50.28 ± 4.91
Ca (g/kg)	46.61 ± 2.50	43.12 ± 2.49	42.19 ± 4.13	40.31 ± 3.70	55.44 ± 4.52
Mg (g/kg)	19.47 ± 0.27	19.73 ± 0.29	19.94 ± 1.20	19.54 ± 1.39	20.95 ± 0.93
Na (g/kg)	9.414 ± 0.215	9.333 ± 0.093	12.539 ± 1.894	10.918 ± 0.695	11.605 ± 0.670
Fe (g/kg)	41.69 ± 0.52	41.31 ± 0.60	38.66 ± 1.57	41.32 ± 1.49	33.51 ± 1.47
Zn (mg/kg)	79.75 ± 8.62	76.27 ± 3.04	60.10 ± 4.89	68.42 ± 6.43	75.45 ± 7.96
Cr (mg/kg)	71.67 ± 3.76	65.83 ± 3.42	74.81 ± 6.85	80.70 ± 17.89	59.91 ± 0.85
Cu (mg/kg)	32.11 ± 0.61	32.21 ± 0.85	29.41 ± 0.95	29.34 ± 1.68	25.66 ± 0.75
Ni (mg/kg)	36.67 ± 1.09	35.73 ± 0.75	45.86 ± 4.29	57.90 ± 9.88	29.69 ± 1.00
Pb (mg/kg)	20.57 ± 0.89	19.93 ± 0.86	21.84 ± 1.44	20.94 ± 2.27	19.74 ± 1.63
As (mg/kg)	14.60 ± 0.95	13.68 ± 0.46	13.04 ± 1.25	11.43 ± 0.97	12.93 ± 0.38
Se (mg/kg)	0.32 ± 0.03	0.30 ± 0.02	0.20 ± 0.03	0.20 ± 0.03	0.28 ± 0.02
Hg (mg/kg)	0.12 ± 0.03	0.145 ± 0.04	0.12 ± 0.04	0.12 ± 0.05	0.06 ± 0.01

**Figure 2:**
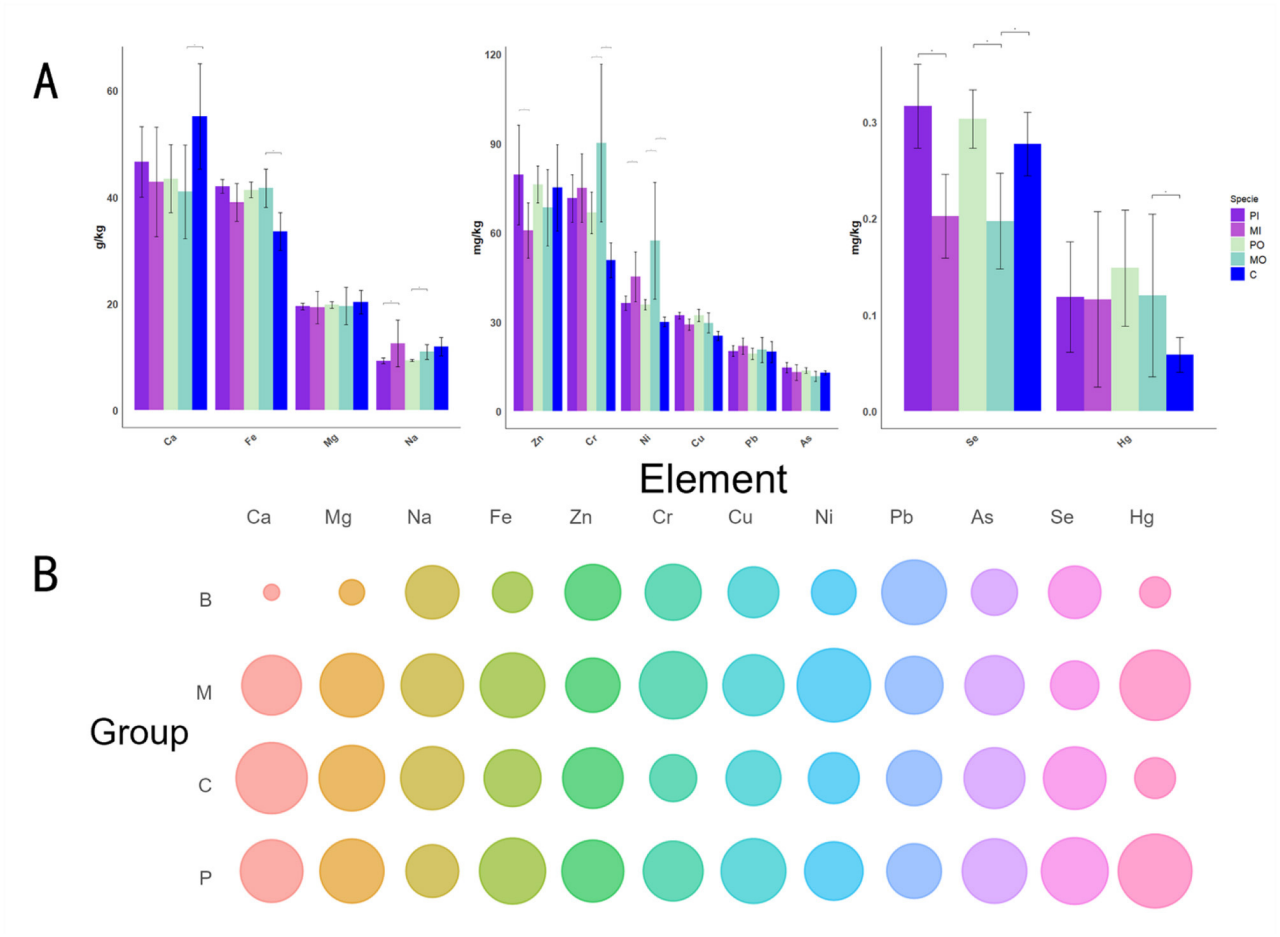
**(A)** The *t*-test (*p < 0.05) was utilized to examine the differences for each individual indicator among 12 elements in histogram. To enhance the clarity and intuitiveness of the figures, the order of the bar chart groups was rearranged to facilitate comparisons between adjacent pairs. Additionally, to investigate the unique soil properties of the marmot groups, only the differences among 3 specific pairs (PI and MI; PO and MO; MO and C) were compared; **(B)** The bubble chart was used to display the relative value of 12 element values in different groups, the B, C, M, and P groups correspond to Chinese baseline soil properties, control group soil, marmot group soil, and pika group soil, respectively.

The *t*-test was utilized to evaluate the differences between every 2 groups for each of the 12 elements presented in a histogram, and a bubble chart displayed the relative values of these 12 elements across different groups (Figure 2), with reference to the Chinese baseline soil values (Xiaohuan et al., 2021). The Chinese baseline soil values in this article were derived from the arithmetic mean of soil samples from regions in China with minimal human impact (Those samples include minimal human impact and human impact). These data were collected by the China Geological Survey between 1999 and 2013 over an area of 1.5 million square kilometers. According to the results, it was found that the elements with the highest content were Fe and Ca (Figure 2A). Significant differences (*p* < 0.05) were observed in the soil elements of Ca, Fe, Cr, Ni, Se, and Hg between the marmot-soils and the control group soils (Figure 2A). Specifically, marmot-soils had elevated levels of Fe, Cr, Ni, and Hg compared to the control soil, while showing lower levels of Ca and Se (Figure 2A). The differences between these values are generally around 20%. Additionally, notable variations in element concentrations were detected between marmot-soils and pika-soils. In detail, the marmot-soils had higher concentrations of Na, Cr, and Ni than pika-soils, both inside and outside of burrows, while Zn and Se showed the opposite trend (Figure 2A). The bubble chart revealed that the levels of elements Ca, Mg, and Fe in the epidemic foci areas were significantly higher than the background values (Figure 2B). Furthermore, the differences were quite significant, as the values of these indicators in rodent habitats were typically more than 1 time higher than the Chinese baseline soil values. The marmot group had obviously higher levels of Cr and Ni compared to other groups, whereas Se and Zn exhibited an opposite trend (Figure 2B).

### Differences in microorganism of soil between different groups

**Figure 3:**
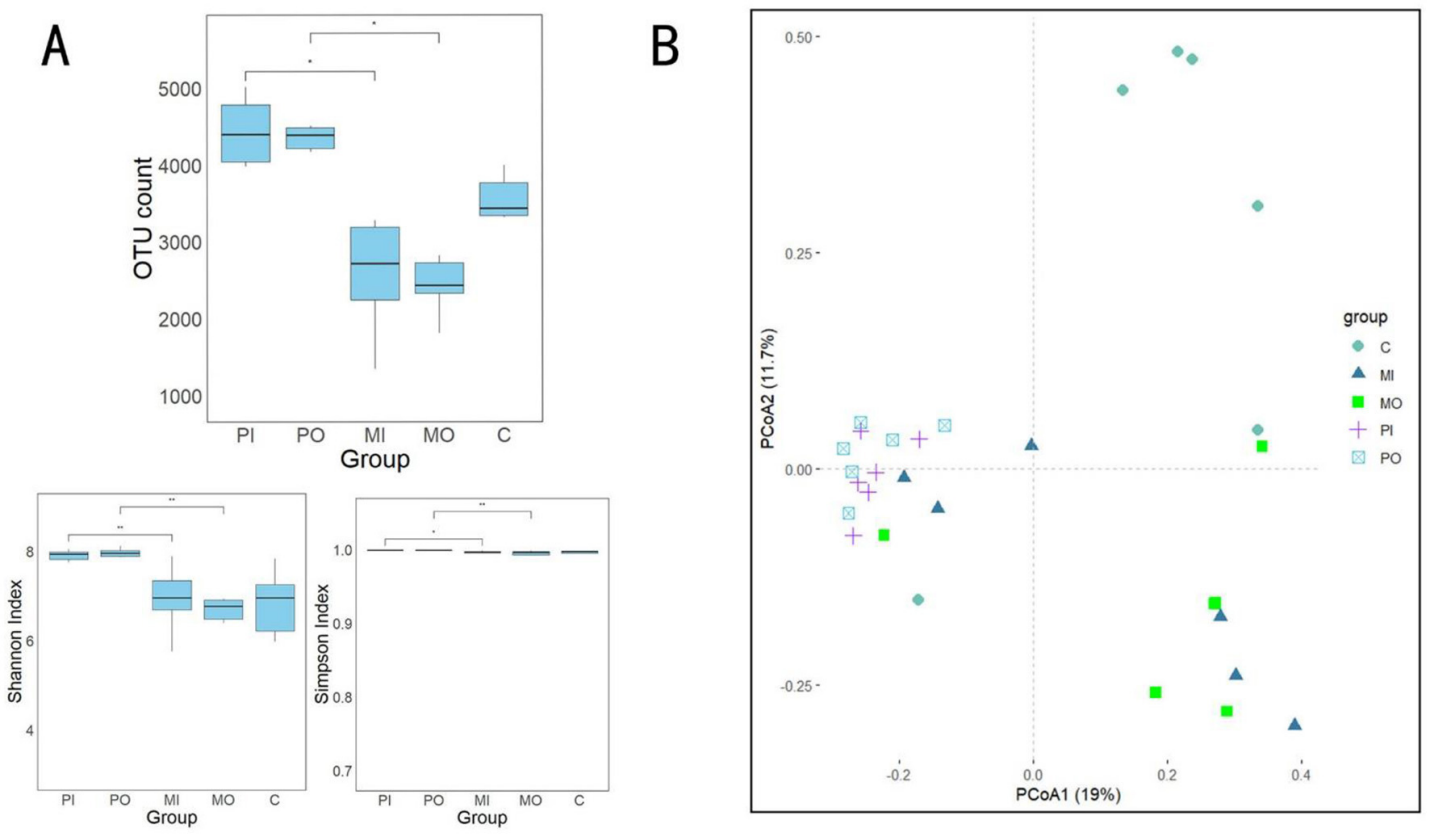
**(A)** Analysis results of soil bacterial alpha diversity among different groups, a total of 3 indices were calculated here: OTU count, Shannon index and Simpson index (*t-*test, *p < 0.05); **(B)** principal coordinate analysis (PCoA) based on Bray-Curtis distance was used to visualize the differences in bacterial community structures among different groups.

The results of the alpha diversity analysis and principal coordinate analysis (PCoA) for 30 soil samples were presented (Figure 3). According to this, significant differences (*p* < 0.05) were observed between soil samples from the marmot groups and the pika groups in 3 indices: OUT count, Shannon index, and Simpson index (Figure 3A). Specifically, compared to the pika group, these indices were significantly lower in the marmot group, with the OTU count being approximately 1,000 lower than the control group and around 2,000 lower than the pika group (Figure 3A). These indices indicate that the soil within marmot habitats had the lowest alpha diversity. It was discovered that there existed a certain degree of difference in bacterial communities between the soils inside and outside marmot burrows, with a marmot outside burrow group and a control group featured unique bacterial communities (Figure 3B). The bacterial communities in marmot groups displayed dichotomy, with one segment closely resembled the bacterial community structure of the pika groups and another showed significant dissimilarity from the bacterial communities of other groups (Figure 3B). Conversely, the pika groups' soil bacterial community structure appeared more homogeneous, while the control group presented a broader variability in their soil bacterial community structure (Figure 3B).

**Figure 4:**
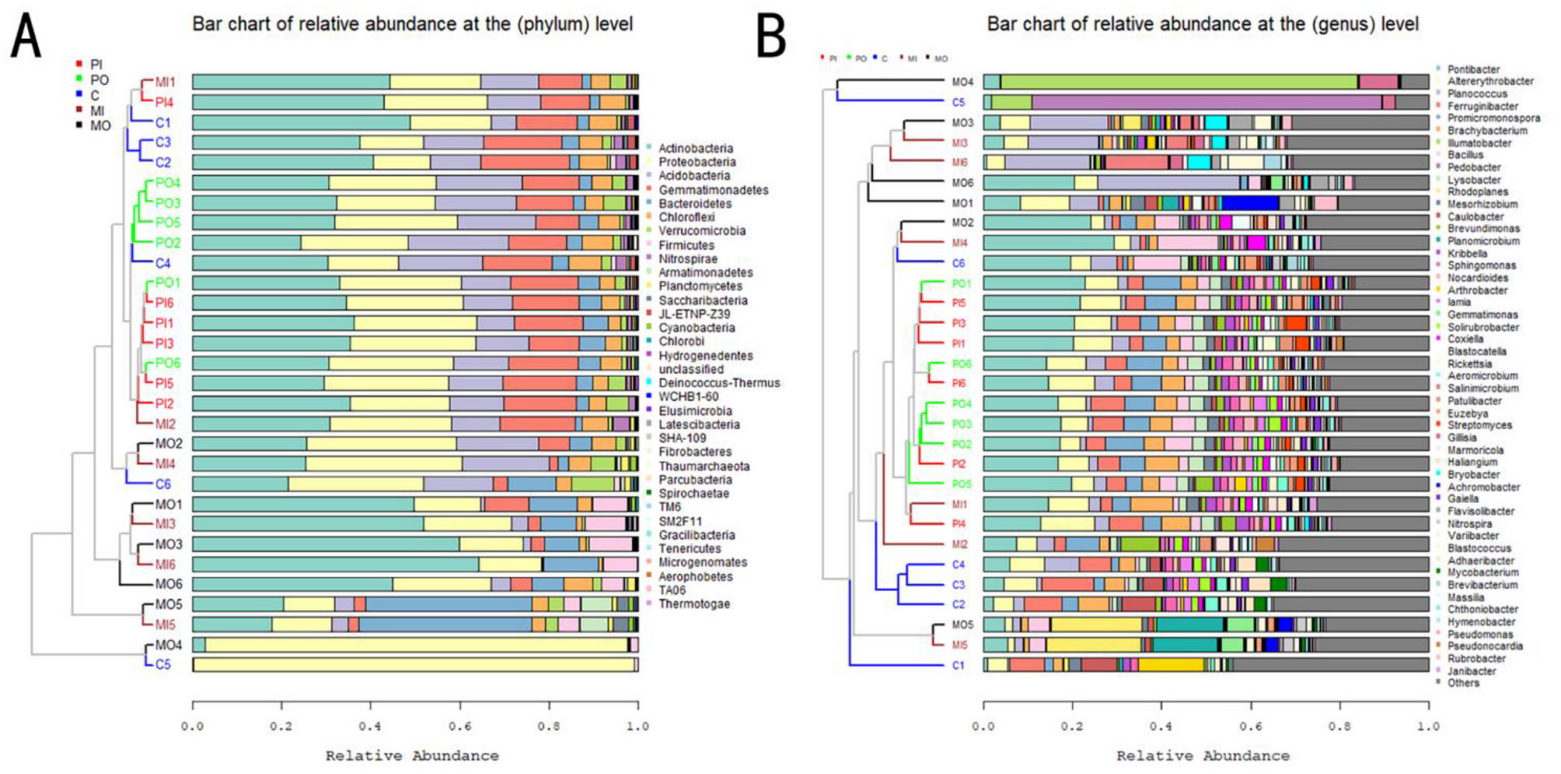
**(A)** The stacked bar charts were constructed based on the relative abundance of bacterial phyla; **(B)** the stacked bar charts were constructed based on the relative abundance of bacterial genera. Due to the large number of bacterial genera, only the top 50 genera with higher relative abundances were presented here, while the remaining genera are collectively represented as “Others.”

The dendrograms to the left of the stacked charts illustrated the similarity among different groups' bacterial abundances and communities, while the bar plots on the right provide detailed information on the relatively dominant bacterial taxa in each group. In the analysis of 16S rRNA amplicon sequencing results, it was found that the inside and outside burrows groups of marmots and pikas exhibited similar bacterial relative abundances, clustering relatively closely (Figure 4). In the taxonomic profile, a total of 33 phyla were identified based on the selection criteria mentioned above, with *Actinobacteria* (34.4% ± 2.3%), *Proteobacteria* (26.2% ± 1.5%), and *Acidobacteria* (12.9% ± 0.7%) had the highest relative abundance percentages, making them the dominant phylum of all soil groups. Additionally, a total of 479 genera were identified, with *Sphingomonas* (14.97% ± 0.96%), *Nocardioides* (7.45% ± 1.16%), and *Arthrobacter* (7.30% ± 1.45%) had the highest relative abundance percentages, making them the dominant genera of all soil groups. However, it was observed that there were some distinctive soil bacterial structures in marmot habitats, such as the MO4 group, which contained more *Proteobacteria* (phylum) and *Coxiella* (genus), respectively, while the MI4 and MO5 groups harbored higher abundances of *Bacteroidetes* or *Salinimicrobium* and *Gillisia*, respectively (Figure 4). At the phylum level, the contents of *Actinobacteria, Bacteroidetes*, and *Firmicutes* in the soil of marmot habitats were all higher than those in other groups, with relatively obvious differences (Figure 4A). At the genus level, the contents of *Arthrobacter* and *Coxiella* in the soil of marmot habitats were higher than those in other groups, while the contents of *Iamia* and *Solirubrobacter* were lower than those in other groups (Figure 4B).

### The influence of soil physicochemical properties on microorganisms

**Figure 5:**
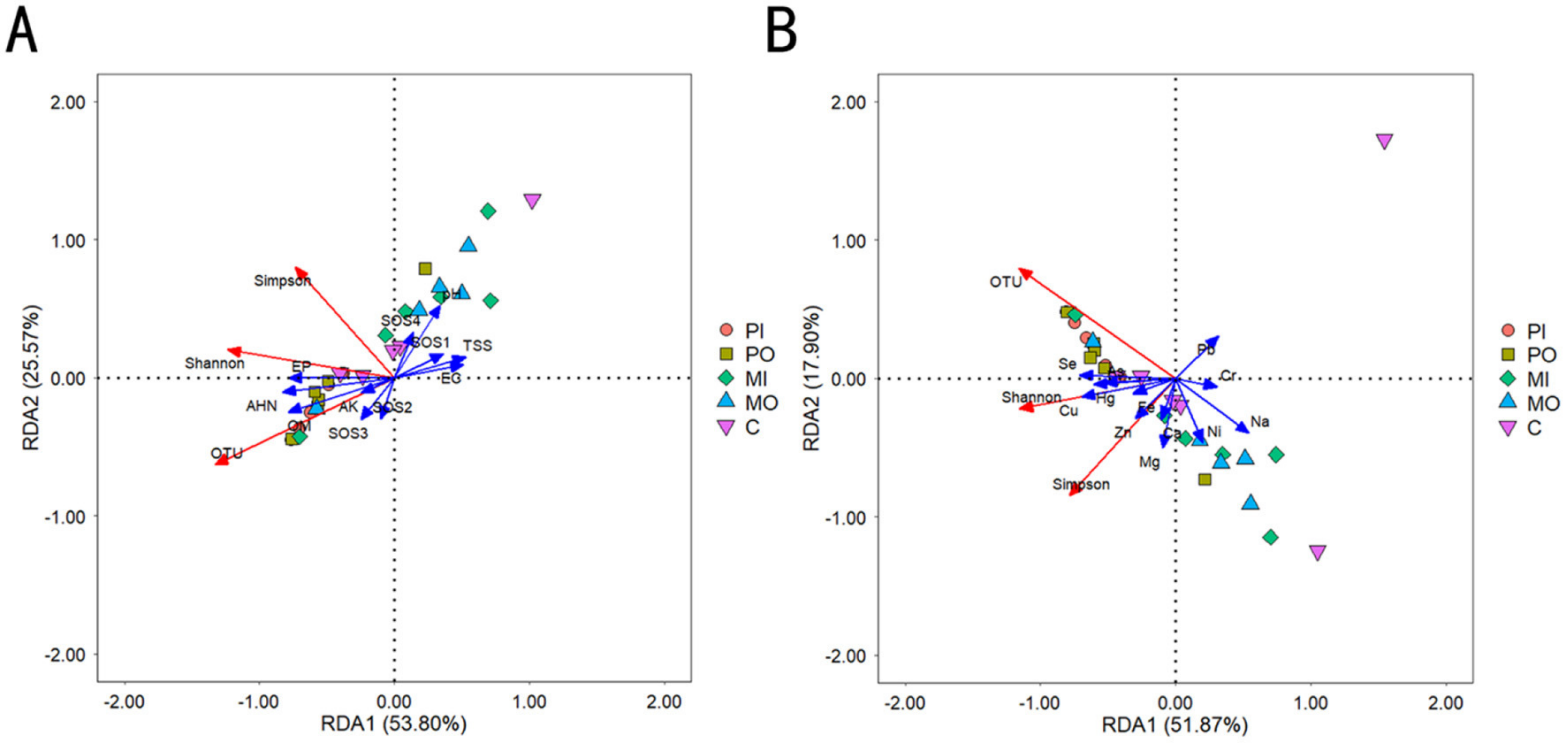
**(A)** Redundancy analysis between soil microbial community and soil first 8 physicochemical properties; **(B)** redundancy analysis between soil microbial community and soil elements.

The structure of the microbial community was significantly influenced by environmental factors. The results of the Redundancy Analysis (RDA) could reflect the relationship between community structure and environmental variables, thereby revealing important environmental drivers affecting the distribution of microorganisms (Figure 5). The cumulative explanation of 79.37% of the variation in soil microbial alpha diversity by the first and second ordination axes was caused by changes in the content of the first 8 soil physicochemical properties. The results indicated that EP, OM, and AHN had a positive impact on soil microbial alpha diversity, whereas EC and TSS had an opposite effect (Figure 5A). Similarly, the cumulative explanation of 69.77% of the variation in soil microbial alpha diversity by the first and second ordination axes was caused by changes in soil element content. The study's findings showed that Fe, Cu, Zn, Hg, Se, and As exerted a positive influence on soil microbial alpha diversity, whereas Pb, Cr, Na, and Ni had a contrasting effect (Figure 5B).

## Discussion

This study comprehensively investigated soil characteristics within the habitats of two plateau animals living in adjacent regions of the Qilian Mountains, using local rodent-free areas and Chinese baseline soil values as controls. It was found that the physicochemical properties and microorganisms of the soil in the marmot habitats exhibited significant differences compared to other groups. In this study, the soil pH values in the region were generally above 8, a condition attributed to the influence of the local climate and geographical factors. Generally speaking, they tended to alkalize the shallow soil (0–10 cm) and alpine grassland soil (Sun et al., 2023). Data revealed that TSS and EC in the soil of marmot habitats were significantly higher than those in pika habitats, with values differing by up to 10-fold, and both were lower than TSS and EC in the control group soil. This indicated that these soil characteristics were specific to the marmot habitats. A study in the United States analyzed a dataset and concluded that plague epidemics were significantly correlated with high soil salinity (electrical conductivity > 4,000 us/cm) and an aridity index < 0.5, which are typical of arid or semi-arid regions (Barbieri et al., 2020). Similarly, in the plague-endemic area of the Qilian Mountains investigated in this study, the soil in the marmot habitats, rather than the pika habitats, exhibited these characteristics. This suggested that TSS and EC might be a clue that plague prevalent in marmot.

In this study, the OM exhibited the same trend as AHN, with soil in marmot habitats presented lower values 2 times than those found in both pika habitats and the control group. Existing studies demonstrated that, compared to control areas, the OM and AHN values in the soil surrounding marmot burrows were lower (Whitesides, 2015). In contrast, it was found that the OM and AHN values of the soil around the pika burrows were similar those of the control group. A study had explained that the digging activities of pikas rarely resulted in a decrease in vegetation biomass. Concurrently, these digging activities brought deep soil containing OM and AHN to the surface, thus having little impact on the OM and AHN values in the soil inside and outside of the pika burrows (Qin et al., 2021). In contrast, marmot digging activities transported soil with a higher proportion of sand or gravel, which contained less organic matter, from deeper layers to the finer-textured surface soil. This increased the soil particle size and subsequently reduced the levels of OM and AHN (Whitesides and Butler, 2016; Yoshihara et al., 2010). Soil with a suitable proportion of large particles exhibited excellent porosity, which ensured good air circulation within the burrows and contributed to the formation of a stable support structure. As a result, the soil inside marmot burrows contained the highest proportion of coarse sand (Warren et al., 2017).

Redundancy analysis indicated that soil OM and AHN values were positively correlated with soil alpha diversity, while EC and TSS showed the opposite trend. This coincidence was indicated that soil OM and AHN might have played a role similar to that of soil alpha diversity in influencing the prevalence of plague. Relevant studies had demonstrated that soil bacterial alpha diversity could influence the prevalence of plague (Abdel et al., 2023). Generally, the diversity of soil microorganisms in the natural plague foci was low, and *Yersinia pestis* could coexist with other microorganisms in soils with low abundance of total microbial species. In this study, soil OM, AHN values, and bacterial alpha diversity were found to be the lowest in marmot habitats compared to other groups. This specificity suggested that these conditions were conducive to the survival of *Yersinia pestis*.

There appeared to be a connection between the natural plague foci and certain metal elements, many of which were crucial for key physiological activities of bacteria, such as increased pathogenicity and biofilm formation (Perry et al., 2015). In the plague foci of marmots, the levels of Ca, Fe, Mg, and Na were the highest, all surpassing Chinese baseline soil values. This suggested that these 4 elements might be closely associated with the plague. Experts from the Chinese Academy of Sciences found that plague origins were often linked to soil rich in Ca and Fe (Wei et al., 2006). In marmot habitats, Ca and Fe in the environment were essential for the *Yersinia pestis* and were closely related to its virulence and growth (Fetherston et al., 2010; Schneewind, 2016). The ability of *Yersinia pestis* to survive in high-salt environments might have contributed to a certain degree of host selection, as the Na content within pika habitats was lower.

In the last century, it had been discovered that soil could harbor bacteria and transmit diseases, with the incidence and severity of diseases influenced by the characteristics and components of the soil (Weinberg, 1987). In this paper, compared to other groups, the marmot habitats soil contained the lowest levels of Se and Zn, while the content of Cr and Ni were the highest. The amount of minerals in soil had the capacity to inhibit or enhance host defenses, and Se and Zn had been shown to play a role in animals' defense mechanisms to against certain diseases. Consequently, the decreased levels of Se and Zn could potentially elevated the risk of plague outbreaks among marmots (Kosoy and Biggins, 2022). An increase in the Ni content in soil had been shown to favor the infection of *Yersinia pestis* (Malek et al., 2017). The redundancy analysis between soil elements and alpha diversity revealed that the elements Zn and Se were positively correlated with alpha diversity, whereas there was a negative correlation with Ni. Previously, we discussed that low alpha diversity in soil could facilitated the infection of *Yersinia pestis*, and here, the soil environments with low levels of Zn and Se and high levels of Ni were found to have the same direction of impact on the survival of *Yersinia pestis* as the aforementioned alpha diversity. Their relationship indirectly supported our view, rather than a conflict, they exhibited synergy.

Analysis of different groups within the natural plague foci revealed significant differences in soil microorganisms, including soil inside and outside marmot burrows. This suggested that soil microorganisms would show obvious differences when influenced by different types of rodents, and the influence of marmots on soil also manifested great differences. The soil in marmot habitats was abundant in *Actinobacteria, Bacteroidetes*, and *Firmicutes*, with their abundances obviously higher than those in the control group and the pikas group. Studies had indicated that the fleas within the plague foci exhibit relatively high abundances of *Actinobacteria, Bacteroidetes*, and *Firmicutes* (Jones et al., 2015). Although these 3 dominate bacterial groups had been proven to be prevalent in the bacterial communities of other vectors, and the bacterial composition of soil was also dominated by these 3 groups (Andreotti et al., 2011; Colman et al., 2012). However, the coincidence that the main microbial groups in both the soil and fleas from the natural plague foci include these 3 bacterial groups might indicate some relationship between soil microbial colonies, flea microbiomes, and the prevalence of plague, which needed future study. An infection experiment was conducted on fleas using *Yersinia pestis* and compared the bacterial communities of fleas before and after infection (Jones et al., 2013). It was discovered that the bacterial communities within infected fleas underwent notable changes, with a marked decrease in *Firmicutes* abundance and a near-complete disappearance of *Bacteroidetes* in control fleas, indicating that *Yersinia pestis* could alter the flea-associated bacterial community in an unknown manner (Jones et al., 2013). Therefore, *Bacteroidetes* and *Firmicutes* in the soil might played a relevant role in the survival of *Yersinia pestis* in the soil and in the process of flea infection with plague. Some form of inhibition or exclusion might have existed between them, but further research was needed to uncover their potential roles in plague transmission.

In this study, the soil samples outside a marmot burrow group and a control group were found to contain a diverse range of bacteria belonging to the *Coxiella*, exhibiting relatively high abundances. These bacteria, capable of transmitting Q fever, had been proven to survive in soil and propagate among rodents (Lu et al., 2022; van der Hoek et al., 2011). This finding indicated that Coxiella survival could present in the soil of plague foci, or certain organisms associated with marmots might have been hosts for Coxiella, as no studies reported the isolation of Coxiella from marmots. Additionally, a significant quantity and variety of *Rickettsia*, including *Rickettsia felis*, were detected in the same control group samples, also indicating contamination by relevant vector organisms (Kuo et al., 2012).

## Conclusion

This study revealed the distinctive soil characteristics of plateau animal marmot habitats, and found an association between the transmission of plague and these characteristics. These results emphasized the distinctive features of marmot-soils in relation to the survival and infection of *Yersinia pestis*, as well as their roles in these processes. These findings contributed to understanding the important role of soil in plague dynamics and provided a new perspective for analyzing the reason of plague prevalent. Further research was needed to strengthen and confirm the relationship between plague prevalence and soil characteristics.

## Data Availability

The raw data supporting the conclusions of this article can be found in online repositories. The name of the repository and accession number can be found below: National Center for Biotechnology Information (NCBI) BioProject, https://www.ncbi.nlm.nih.gov/bioproject/, PRJNA1171125.
